# Menthol‐like cooling compounds, including (R)‐(‐)‐carvone, inhibit the human bitter taste receptors for saccharin and acesulfame K

**DOI:** 10.1002/2211-5463.70098

**Published:** 2025-08-06

**Authors:** Miyuu Saito, Takumi Misaka

**Affiliations:** ^1^ Department of Applied Biological Chemistry, Graduate School of Agricultural and Life Sciences The University of Tokyo Japan

**Keywords:** aftertaste, artificial sweetener, cellular assay, taste receptor, TRPM8

## Abstract

G protein‐coupled receptors (GPCRs) are responsible for sensing sweet, umami, and bitter tastes. Bitter taste receptors belong to the taste receptor type 2 (TAS2R) family, and although trigeminal stimulants, such as menthol, have been reported to reduce bitterness, little is known about whether and how they affect the function of TAS2R. Here, we report that some menthol‐like cooling compounds, including (R)‐(‐)‐carvone, act as inhibitors of TAS2R31 and TAS2R43, which are taste receptors responsible for the intrinsic bitter aftertaste of saccharin and acesulfame K. Since (R)‐(‐)‐carvone only exerted a faint cooling effect and a cooling effect is often not preferred in food flavor design, this compound is expected to be highly effective in improving the unpleasant aftertaste of artificial sweeteners. Thus, this study not only provides novel insights into the mechanism by which trigeminal nerve stimulants improve the aftertaste of artificial sweeteners but also useful information for the flavor design of future food products containing artificial sweeteners.

## Graphical abstract

We report that some menthol‐like cooling compounds, including (R)‐(‐)‐carvone, act as inhibitors of TAS2R31 and TAS2R43, which are taste receptors responsible for the intrinsic bitter aftertastes of saccharin and acesulfame K. However, there was little correlation between the intensity of the cooling sensation and the potency of bitterness inhibition.

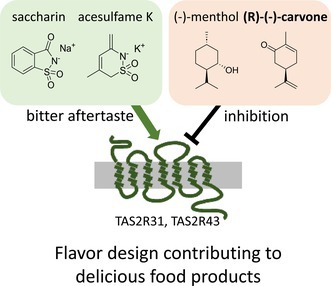

**Abbreviations**
AUC: area under the curve
DMSO: dimethyl sulfoxide
GPCR: G protein‐coupled receptor
HEK293T cells: human embryonic kidney 293T cells
RFU: relative fluorescence units
SEM: standard error of the mean
TAS2R: taste receptor type 2

Taste is the chemical sensation that occurs due to the presence of substances in the oral cavity. These chemical sensations play an important role in making appropriate choices for survival by promoting the ingestion of nutritious foods and avoiding the ingestion of toxic foods. Humans have five basic tastes: sweet, umami, salty, sour, and bitter [1]. Many substances used as energy sources and for protein synthesis, such as sugars and amino acids, are palatable, exhibiting sweetness and umami, thus promoting feeding [2]. In contrast, repellent substances, such as poisons and those that are putrefied, exhibit aversive tastes, such as bitterness and sourness, to inhibit feeding [3, 4]. Taste substances are received by taste receptors expressed on taste cells that comprise the taste buds and elicit a taste sensation. G protein‐coupled receptors (GPCRs) are responsible for sensing sweet, umami, and bitter tastes, among which bitter taste receptors belong to the taste receptor type 2 (TAS2R) family [5, 6, 7]. Humans have 26 types of TAS2Rs [8], and their diversity enables the recognition of a wide range of bitter substances [9].

Artificial sweeteners are known to have an intrinsic bitter aftertaste [10, 11]. Artificial sweeteners are widely used in reduced‐calorie foods and beverages worldwide [12], but their unpleasant aftertaste adversely affects consumer acceptance [10]. Therefore, overcoming these negative aftertastes is a challenge for food product development. Saccharin and acesulfame K are two typical artificial sweeteners that present a bitter aftertaste, which was discovered in the 1950s [13, 14, 15]. Since then, these sweeteners have been evaluated using sensory tests, and strategies for mitigating their unpleasant taste have been investigated. These strategies include combining them with other sweeteners [16, 17] or cold substances [18].

After the discovery of bitter taste receptors in the 2000s, it became clear that these unpleasant bitter aftertastes were perceived via two types of bitter taste receptors, TAS2R31 and TAS2R43 [19]. The identification of corresponding bitter taste receptors has demonstrated that inhibitors of these receptors can mitigate the bitter aftertaste associated with artificial sweeteners, prompting ongoing research in this area [20, 21]. For instance, in 1955, cyclamate was found to improve unpleasant tastes through sensory evaluation [16], and in 2017, it was confirmed to act as an inhibitor of TAS2R31 and TAS2R43 by the taste receptor assays [21]. Additional inhibitors of TAS2R31 and TAS2R43, such as GIV3727, have also been documented [19]. However, the use of these inhibitors in food products is impractical because GIV3727 is a synthetic compound that is expensive to generate, whereas cyclamate, an artificial sweetener, has a strong sweet taste that affects the taste profile of the products. Therefore, we aimed to identify new inhibitors of TAS2R31 and TAS2R43 that are suitable for food use and have less of an impact on taste design.

In this study, we investigated the inhibitory effects of typical trigeminal stimulants on human bitter taste receptors for use with artificial sweeteners, since it has been reported that trigeminal stimuli can modulate bitterness [22, 23, 24]. Recently, it was reported that mixing with menthol improved the aftertaste of acesulfame K [18]. However, these findings were based on human sensory tests, and the mechanisms underlying these phenomena have not been examined, especially regarding the interactions between the taste receptors and these chemical compounds. Therefore, in this study, we investigated whether typical trigeminal stimulants such as menthol suppress bitter tastes by inhibiting bitter taste receptors using cellular assays. These results provide new insights into the taste design of foods that contain artificial sweeteners.

## Methods

### Reagents

The ligands were diluted in assay buffer (10 mm 4‐[2‐hydroxyethyl]‐1‐piperazineethanesulfonic acid (HEPES), 130 mm NaCl, 10 mm glucose, 5 mm KCl, 2 mm CaCl_2_, and 1.2 mm MgCl_2_ [pH adjusted to 7.4 with NaOH]) to the desired concentrations. The hydrophobic compounds were first dissolved in dimethyl sulfoxide (DMSO) and then diluted in assay buffer to achieve a final DMSO concentration of ≤0.1% (v/v) to avoid cytotoxicity.

### Chemicals

Samples were obtained from commercial sources, as follows: sodium saccharin dihydrate, acesulfame K, menthone, and andrographolide were purchased from FUJIFILM Wako Pure Chemical Corporation (Osaka, Japan); (−)‐menthol was obtained from Kanto Chemical (Tokyo, Japan); 1,8‐cineole was procured from Sigma–Aldrich (Tokyo, Japan); and capsaicin, (+)‐menthol, (R)‐(‐)‐carvone, (R)‐(‐)‐piperitone, (+)‐pulegone, (−)‐menthyl acetate, and (−)‐limonene were obtained from Tokyo Chemical Industry Co. Ltd. (Tokyo, Japan).

### Construction of the expression plasmids

In accordance with a previous report [25], each TAS2R gene was tagged at the N terminus with the signal sequence of the rat muscarinic acetylcholine receptor (M3). The NM_024080.5 nucleotide sequence was used for TRPM8. The genes were subcloned and inserted into a pEAK10 expression vector (Edge Biosystems, Gaithersburg, MD, USA). Construction of the expression plasmid for apophotoprotein (mt‐apoclytin II) [26] and the chimeric G protein α subunit Gα16gust44 [27] has been reported previously.

### Cell culture and transfection

HEK293T cells were cultured in Dulbecco's modified Eagle's medium (Sigma–Aldrich, Tokyo, Japan) supplemented with 10% fetal bovine serum (Thermo Fisher Scientific, Waltham, MA, USA) at 37 °C under 5% CO_2_. Cells were seeded into 6‐well plates and then transiently transfected with the plasmids using Lipofectamine 2000 (Thermo Fisher Scientific).

### Ca^2+^ imaging

The method used for Ca^2+^ imaging was described in our previous report [28]. Briefly, HEK293T cells were transiently transfected with plasmids for 3.2 μg of M3‐TAS2Rs and 0.8 μg of Gα16gust44 together with 0.1 μg of red fluorescent protein (pDsRed2‐N1; Takara Bio, Shiga, Japan). After 6 h, the transfected cells were seeded into 96‐well plates (Lumox multiwell 96‐well, Starstedt AG and Co., Nümbrecht, Germany) at a density of 40 000–50 000 cells/well. After 18–24 h of incubation at 37 °C under 5% CO_2_, the cells were washed with assay buffer and treated with 5 μm Fura‐2‐acetoxymethyl ester (Fura‐2AM; Thermo Fisher Scientific) for 30 min at 27 °C in the dark. The cells were subsequently washed with assay buffer again and incubated for an additional 10 min at room temperature.

Ligands were manually added to the wells in a 100‐μL aliquot of assay buffer containing the ligands at twice the desired final concentration. The fluorescence intensity of Fura‐2 was measured upon excitation at 340 and 380 nm, followed by detection at 510 nm with a Lambda 10–3 computer‐controlled filter changer (Sutter Instrument, San Rafael, CA, USA), a CoolSNAP HQ2 camera (Photometrics, Tucson, AZ, USA), and an IX‐81 inverted fluorescence microscope (Olympus, Tokyo, Japan). The images were recorded at 4 s intervals and analyzed with MetaFluor software (Molecular Devices, Sunnyvale, CA, USA).

### Luminescence assay with bitter taste receptors

The luminescence assay method has been reported previously [26]. Briefly, HEK293T cells were transfected with 2.8 μg of M3‐TAS2Rs, 0.2 μg of Gα16gust44, and 1.0 μg of mt‐apoclytin II plasmids. After 6 h, the transfected cells were seeded into 96‐well plates (clear‐bottom CellBIND surface plates, Corning Inc., Bedford, MA, USA) at a density of 70 000–80 000 cells/well. After 18–24 h of incubation at 37 °C under 5% CO_2_, the cells were washed with assay buffer and treated with 10 μm coelenterazine (Promega, Madison, WI, USA) for 30 min at 27 °C in the dark.

After 20 s of baseline measurements, an aliquot of the assay buffer supplemented with 2× ligand was added, and luminescence was recorded using a FlexStation 3 microplate reader (Molecular Devices) for an additional 100 s. The response from each well was evaluated on the basis of the area under the curve (AUC), and the relative intensity of the response was calculated by normalization to the response with the lowest concentration of inhibitor (defined as 1.0). For IC_50_ value determination, plots of the amplitudes versus concentrations were fitted to the Hill equation by using Clampfit ver.10.4 (Molecular Devices).

### Fluorescence assay with TRPM8

The fluorescence assay method has been reported previously [29]. Briefly, HEK293T cells were transfected with 3.0 μg of TRPM8 plasmid to evaluate the intensity of the cooling sensation. After 6 h, the transfected cells were seeded into 96‐well plates at a density of 60 000–70 000 cells/well. Then, after 18–24 h of incubation at 37 °C under 5% CO_2_, the cells were washed with assay buffer and treated with the FLIPR Calcium 4 Assay Kit (Molecular Devices) for 30 min at 27 °C in the dark.

Changes in fluorescence upon excitation at 485 nm and emission at 525 nm with a cutoff at 515 nm were monitored at 2 s intervals. A 100‐μL aliquot of assay buffer supplemented with 2× ligands was added at 20 s, and the fluorescence intensity was measured for an additional 100 s. The response of each well was evaluated on the basis of the change in relative fluorescence units (∆RFU), which was calculated as (maximum fluorescence value) – (minimum fluorescence value). The data are presented as the means ± standard errors of the means (SEMs).

### Statistical analysis

Differences were evaluated using one‐way analysis of variance followed by Dunnett's test. We considered *P* values of less than 0.05 to indicate statistically significant differences. KyPlot 6.0 (KyensLab Inc., Tokyo, Japan) was used for analysis.

## Results

### Response of TAS2R31 to saccharin Na in the presence of trigeminal stimulants

First, we examined the inhibitory effects of typical trigeminal stimulants on TAS2R31 by Ca^2+^ imaging. We compared the responses of TAS2R31‐expressing cells to 2.5 mm saccharin Na mixed with each trigeminal stimulant. This saccharin concentration was chosen because it is close to the human sensory threshold of bitterness [30]. Four trigeminal stimulants were selected: (−)‐menthol, (+)‐menthol, 1,8‐cineole, and capsaicin. (−)‐Menthol, (+)‐menthol, and 1,8‐cineole were applied at concentrations of 1 mm, whereas capsaicin was used at 0.1 mm because of its solubility.

**Fig. 1:**
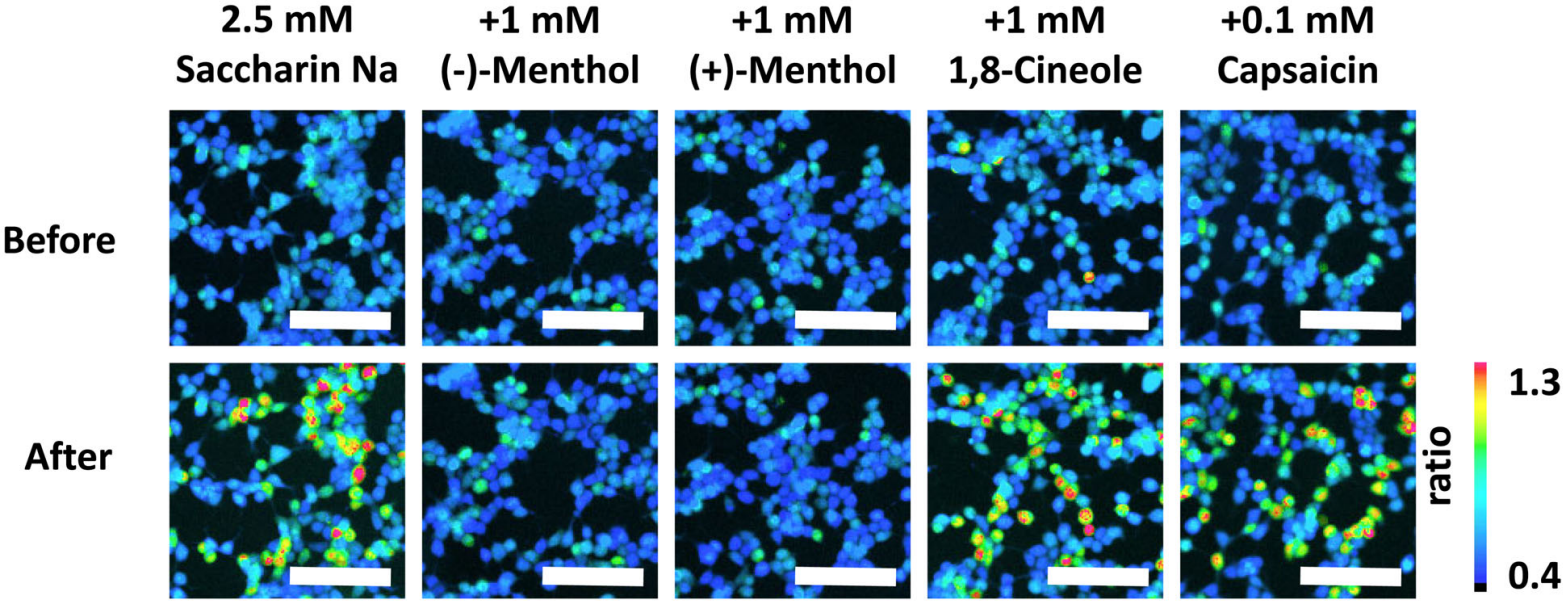
Representative ratiometric images of Ca^2+^ detection in Fura‐2‐loaded HEK293T cells expressing TAS2R31 stimulated with 2.5 mm saccharin Na, in the absence or presence of each trigeminal stimulant. Images were acquired at least three times for each solution, and representative results are shown. The top and bottom columns show representative images of the cells obtained before and after ligand application, respectively. The color scale indicates the pseudocolor of the F_340_/F_380_ ratio. Scale bars: 100 μm.

The addition of (−)‐menthol or (+)‐menthol clearly reduced the responses of TAS2R31‐expressing cells to saccharin Na (Fig. 1). In contrast, the addition of 1,8‐cineole or capsaicin had little or no inhibitory effect. This result showed that not all substances that stimulate the trigeminal nerve inhibit the bitter taste receptor TAS2R31 in the heterologous receptor assay.

### Characterization of the structural features of TAS2R31 inhibitors

**Fig. 2:**
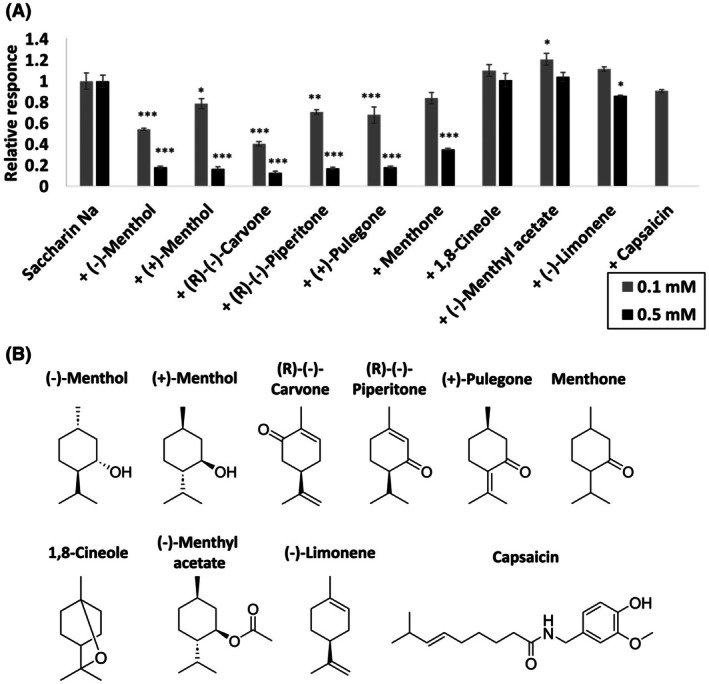
Response of TAS2R31‐expressing cells to 2.5 mm saccharin Na in the presence of menthol‐like compounds. (A) Menthol‐like compounds (0.1 or 0.5 mm) were mixed with saccharin Na (2.5 mm), and a luminescence assay was performed. Each column represents the response relative to that of the control (2.5 mm saccharin Na alone) and is presented as the mean ± SEM (*n* = 6). Significant differences were evaluated using a one‐way analysis of variance followed by Dunnett's test (vs. control: *P < 0.05; **P < 0.01; ***P < 0.001). (B) Chemical structures of the candidate TAS2R31 inhibitors.

Menthols are monoterpene alcohols and representative cooling compounds (Fig. 2). Therefore, the TAS2R31 inhibitory effects of several compounds possessing monoterpene skeletons and different functional groups were examined.

Luminescence assays were used to determine whether the bitter response of TAS2R31 to saccharin Na was inhibited when saccharin Na was mixed with 10 compounds, the structures of which are shown in Fig. 2B. In the luminescence assay, (−)‐menthol and (+)‐menthol significantly inhibited the receptor response, corroborating the Ca^2+^ imaging results (Fig. 1), although the concentrations of menthol used in the luminescence assay were 0.1 and 0.5 mm (Fig. 2). In addition to (−)‐ and (+)‐menthol, (R)‐(‐)‐carvone, (R)‐(‐)‐piperitone, (+)‐pulegone, and menthone showed significant inhibitory effects on TAS2R31, whereas 1,8‐cineole, (−)‐menthyl acetate, (−)‐limonene, and capsaicin had little or only slight inhibitory effects (Fig. 2).

**Fig. 3:**
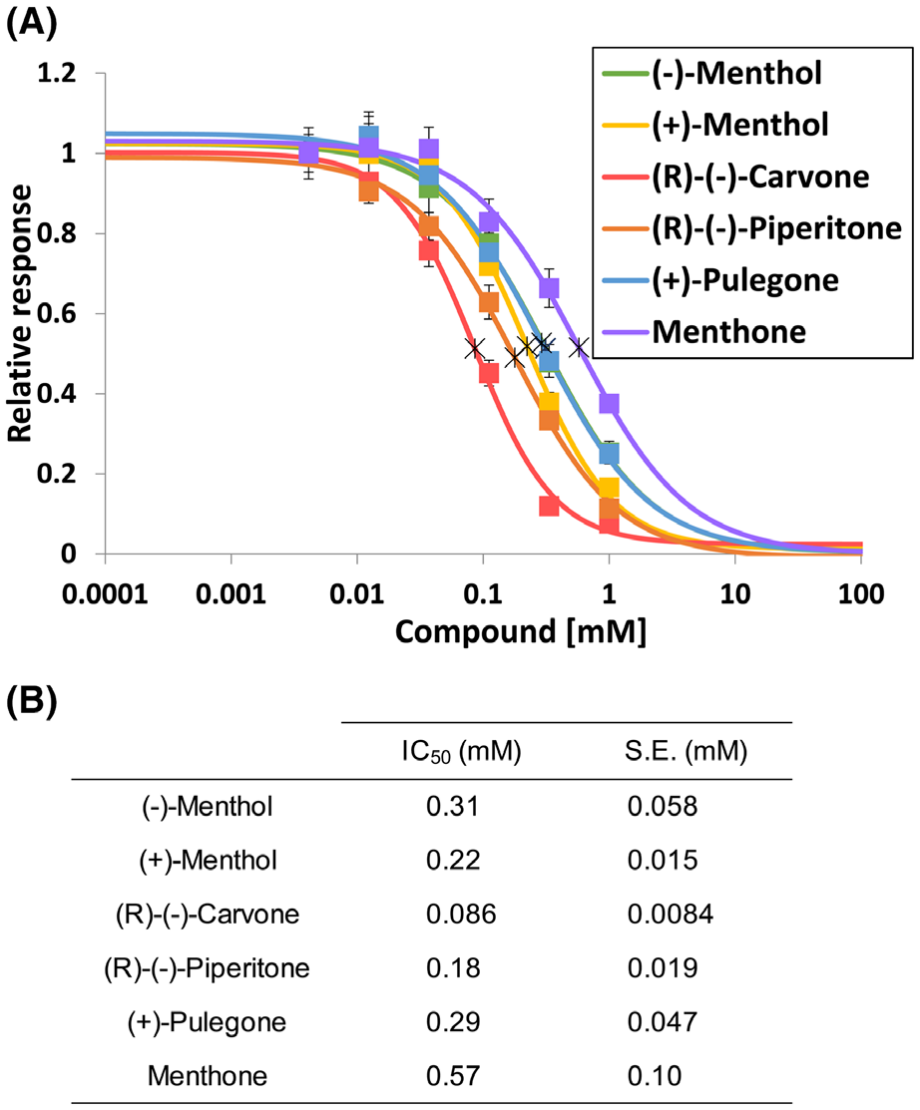
The menthol‐like compounds inhibited the response of TAS2R31‐expressing cells to the saccharin Na in a dose‐dependent manner. (A) The data represent the response relative to that of the lowest concentration of inhibitor (defined as 1.0) and are presented as the means ± SEMs (*n* = 6). The crosses in the graph represent the IC_50_ values. (B) IC_50_ values of each compound.

Inhibition curves were subsequently constructed for the six inhibitors with data obtained from various concentrations of these compounds, and all six compounds showed concentration‐dependent inhibition of TAS2R31 (Fig. 3A). There were slight differences in the inhibitory activity among the compounds tested, among which (R)‐(‐)‐carvone gave the lowest IC_50_ value of 0.086 mm (Fig. 3B). Thus, TAS2R31 was inhibited by some monoterpene alcohols and ketones but not by ethers, esters, or the monoterpene skeleton alone (Fig. 2).

### Inhibitory effects of (R)‐(‐)‐carvone on other bitter taste receptors

As (R)‐(‐)‐carvone was the most potent inhibitor of TAS2R31 among the tested compounds, its inhibition of two other bitter taste receptors, TAS2R43 and TAS2R50, which are homologous to TAS2R31, was also examined. TAS2R43 is the receptor for the intrinsic aftertastes of saccharin and acesulfame K [18]. In addition, we investigated whether (R)‐(‐)‐carvone inhibits the response of TAS2R31 to acesulfame K as well as to saccharin Na. Notably, the response of TAS2R43 to acesulfame K was not the subject of this study because of the weak response (data not shown).

**Fig. 4:**
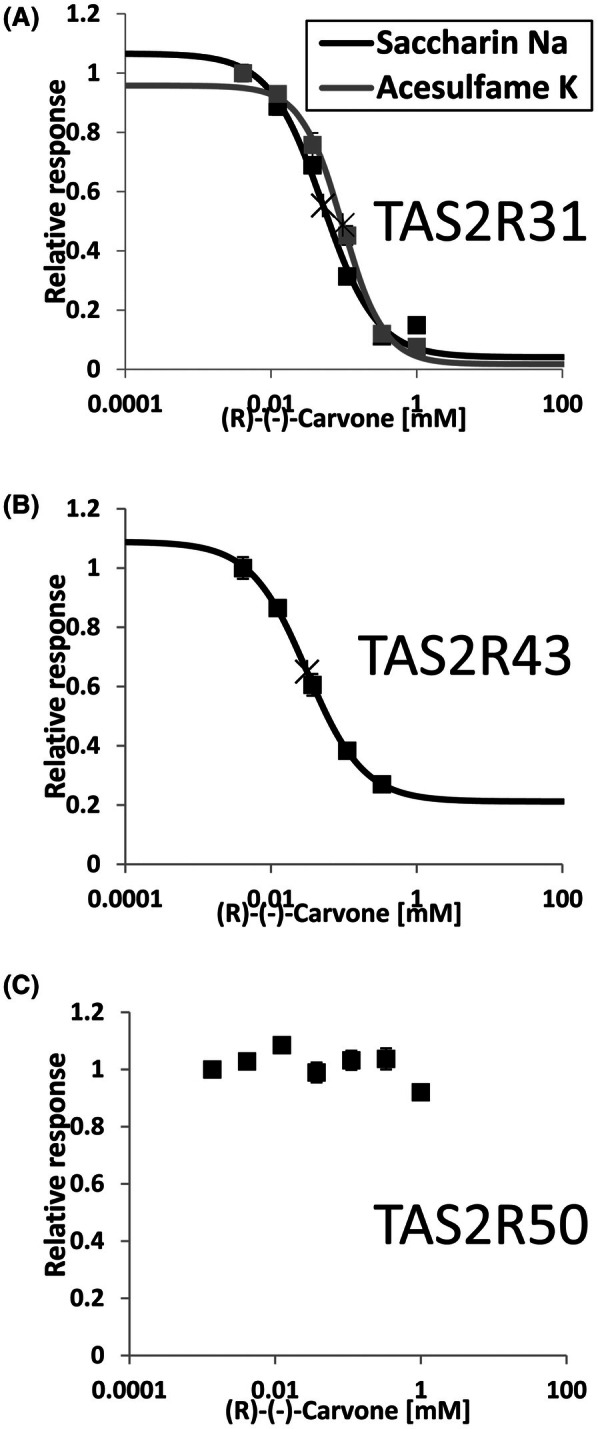
Inhibitory effects of (R)‐(‐)‐carvone on multiple bitter taste receptors. The dose‐dependent inhibitory responses of cells expressing TAS2R31 (A), TAS2R43 (B), and TAS2R50 (C) were examined in the presence of various concentrations of (R)‐(‐)‐carvone. The following ligands were used: 2.5 mm saccharin Na and 5 mm acesulfame K in (A), 2.5 mm saccharin Na in (B), and 3 μm andrographolide in (C). The data represent the response relative to that of the lowest concentration of inhibitor (defined as 1.0) and are presented as the means ± SEMs (*n* = 6). The crosses in the graph represent the IC_50_ values.

The results showed that (R)‐(‐)‐carvone inhibited both TAS2R31 and TAS2R43 (Fig. 4A,B) but not TAS2R50 (Fig. 4C). Furthermore, (R)‐(‐)‐carvone had comparable inhibitory effects on saccharin Na‐ and acesulfame K‐treated TAS2R31 (Fig. 4A). These observations suggest that (R)‐(‐)‐carvone is not a universal inhibitor of all bitter taste receptors but specifically targets and inhibits TAS2R31 and TAS2R43.

### Intensity of the TRPM8 response to menthol‐like compounds

Because (−)‐ and (+)‐menthols are representative cooling compounds and the TAS2R31 inhibitors identified in this study have a menthol‐like structure (Fig. 2B), it was speculated that these inhibitors also induce a cooling sensation. Thus, we performed cold intensity measurements with TRPM8 and 6 menthol‐like inhibitors. 1,8‐Cineole was also used in this assay because it is a known cooling compound that does not inhibit TAS2R31 (Fig. 2).

**Fig. 5:**
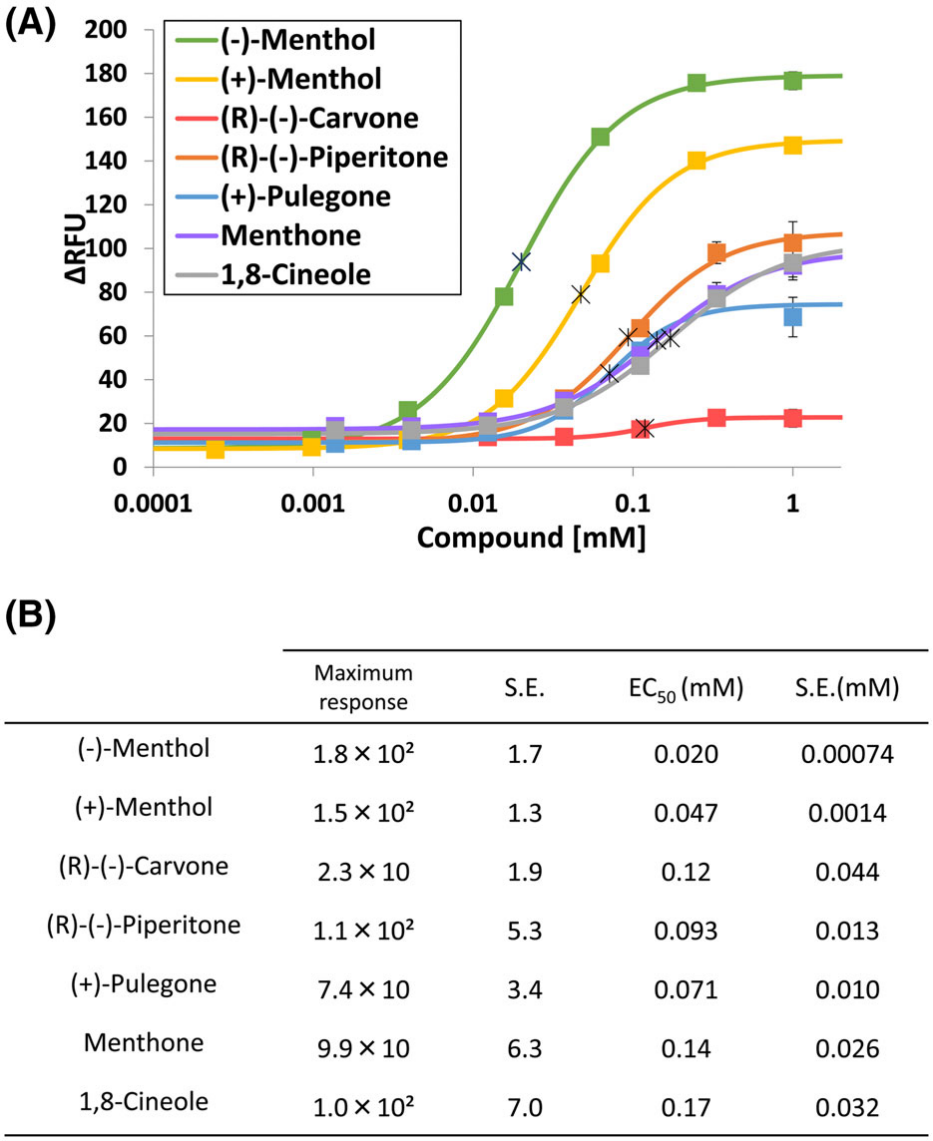
Dose‐dependent responses of TRPM8‐expressing cells to TAS2R31 inhibitors. (A) Dose–response curves were generated for TRPM8‐expressing cells. The data are presented as the means ± SEMs (*n* = 6). The crosses in the graph represent the EC_50_ values. ∆RFU: change in relative fluorescence units. (B) Maximum responses and EC_50_ values for each compound.

The results showed that all substances, except for (R)‐(‐)‐carvone, induced a strong response in TRPM8‐expressing cells (Fig. 5). The substance that produced the strongest cold sensation was (−)‐menthol, followed by (+)‐menthol, while (R)‐(‐)‐piperitone, (+)‐pulegone, menthone, and 1,8‐cineole showed similar cold intensities. These data indicated that most of the identified TAS2R31 inhibitors induce a cooling sensation. However, treatment with (R)‐(‐)‐carvone, which had the greatest inhibitory effect on TAS2R31, led to only a faint cooling sensation (Figs 3 and 5). In addition, (−)‐menthol, which had the highest cold intensity, showed relatively low inhibitory activity against TAS2R31 among the compounds tested (Figs 3 and 5). These results indicated that there was little correlation between the intensity of the cooling sensation or trigeminal nerve stimulation and the TAS2R31 inhibitory potency, although multiple cooling compounds are inhibitors of TAS2R31.

## Discussion

In this study, we investigated inhibitors of the intrinsic bitter aftertaste of artificial sweeteners with a focus on substances that induce trigeminal stimulation, as trigeminal stimulation is known to modulate bitterness. Here, we revealed that some menthol‐like cooling compounds function as inhibitors of TAS2R31 and TAS2R43. In previous sensory tests, trigeminal stimulants were thought to improve bitter taste by stimulating the trigeminal nerve [18, 22, 23, 24]. However, our experiment revealed that some of the cooling compounds actually function as bitter aftertaste blockers that directly interact with bitter taste receptors (Figs 2 and 3) independent of trigeminal stimulation. This study led to new insights into the mechanism by which trigeminal nerve stimulants improve the aftertaste of artificial sweeteners, particularly saccharin and acesulfame K.

An investigation of the inhibitor structures suggested that TAS2R31 inhibitors feature a monoterpene backbone and hydroxyl or carbonyl groups (Fig. 2). Notably, the series of bitter taste inhibitors identified in this study are not structurally similar to any known TAS2R31 or TAS2R43 agonists [7] or their competitive inhibitors GIV3727 [31] and cyclamate [21]. Interestingly, both menthol isomers inhibited these receptors (Figs 1 and 2), whereas only one enantiomer inhibits other taste receptors [32]. The diversity of inhibitor structures suggests that there is a strong inhibitor with a monoterpene backbone and hydroxyl or carbonyl groups and that substances partially structurally similar to the inhibitor exhibit broad and weak inhibitory effects. Of the substances tested in this study, the one most structurally similar to the potent inhibitor is likely to be (R)‐(‐)‐carvone, which exhibited the lowest IC_50_ value; however, at this stage, it is difficult to predict its exact structure. Assuming that it is a natural substance, it is unnatural that it has not been reported because of its potent inhibitory effects. Therefore, the hypothesized potent TAS2R31 and TAS2R43 inhibitors are likely to be naturally occurring or artificial substances rarely found in nature, making their discovery and practical application difficult.

In addition, our results strongly indicate that there is little correlation between the strength of the TAS2R31 inhibitory effect and the intensity of the cold sensation, although some cooling compounds have been shown to inhibit bitter tastes (Figs 3 and 5). This is probably due to the structural similarities among the inhibitors and TRPM8 agonists. In this study, the TAS2R31 inhibitors possessed a monoterpene skeleton with hydroxyl or carbonyl groups. TRPM8 agonists, on the other hand, are characterized by the presence of an isopropyl group and a hydroxyl or carbonyl group, which is consistent with the structure of (−)‐menthol, with some exceptions, such as 1,8‐cineole and icilin [33]. These features of TRPM8 agonists were confirmed in the present study. Additionally, the findings of this study show that there is some structural similarity among the TAS2R31 inhibitors and some TRPM8 agonists, suggesting that some menthol‐like cooling compounds inhibit bitter sensations in a trigeminal stimulation‐independent manner.

## Conclusions

The inhibitory effects of trigeminal stimulants, which have been shown to modulate bitterness in human sensory tests, on bitter taste receptors were investigated to identify inhibitors of the intrinsic aftertaste of artificial sweeteners, such as saccharin and acesulfame K. In this study, we found that some menthol‐like cooling compounds inhibited TAS2R31 and TAS2R43. Among the bitter taste inhibitors identified in this study, (R)‐(‐)‐carvone, a compound with weak cooling effects, exhibited the highest inhibitory activity against TAS2R31. In addition, (R)‐(‐)‐carvone directly inhibited TAS2R31 and TAS2R43 in the presence of both saccharin and acesulfame K. Because a cooling sensation is often not preferred in food flavor design, (R)‐(‐)‐carvone, which exerts only a faint cooling effect, is expected to be highly effective in improving the unpleasant aftertaste of artificial sweeteners. These findings provide useful information for the flavor design of future products containing artificial sweeteners.

## Conflict of interest

The authors declare no conflict of interest.

## Author contributions

TM designed the overall research concept. MS collected and analyzed the data. MS and TM wrote the manuscript.

## Data Availability

The data that support the findings of this study are available from the corresponding author [amisaka@g.ecc.u-tokyo.ac.jp] upon reasonable request.
